# Bodyweight loss in predicting neonatal hyperbilirubinemia 72 hours after birth in term newborn infants

**DOI:** 10.1186/1471-2431-13-145

**Published:** 2013-09-21

**Authors:** Wen-Chieh Yang, Lu-Lu Zhao, Yu-Cheng Li, Chi-Hua Chen, Yu-Jun Chang, Yun-Ching Fu, Han-Ping Wu

**Affiliations:** 1 Department of Pediatrics, Changhua Christian Hospital, Changhua, Taiwan, R.O.C; 2 School of Medicine, Chung Shan Medical University, Taichung, Taiwan, R.O.C; 3 Department of Pediatrics, Taipei Tzuchi Hospital, The Buddhist Medical Foundation, Taipei, Taiwan, R.O.C; 4 Department of Pediatrics, Taichung Tzuchi Hospital, The Buddhist Medical Foundation, Taichung, Taiwan, R.O.C; 5 Department of Pharmacy, Taichung Tzuchi Hospital, The Buddhist Medical Foundation, Taichung, R.O.C; 6 Laboratory of Epidemiology and Biostatistics, Changhua Christian Hospital, Changhua, Taiwan, R.O.C; 7 Department of Pediatrics, Taichung Veterans General Hospital, Taichung, Taiwan, R.O.C; 8 Institute of Clinical Medicine, National Yang-Ming University, Taipei, Taiwan, R.O.C; 9 Department of Medicine, Tzu Chi University, Hualien, Taiwan, R.O.C; 10 Department of Pediatrics, Taichung Tzuchi Hospital, No. 88, Sec. 1, Fongsing Rd., Tanzih Township, Taichung, Taiwan, 42743, R.O.C

**Keywords:** Neonate, Jaundice, Hyperbilirubinemia, Dehydration

## Abstract

**Background:**

Severe dehydration is generally believed to be a cause of significant hyperbilirubinemia in newborn babies. This study aimed to analyze the weight loss of healthy term newborn infants at 24, 48 and 72 hours after birth to predict significant hyperbilirubinemia at 72 hours.

**Methods:**

From January 2007 to December 2008, we conducted this retrospective chart review by measuring total bilirubin (transcutaneous and serum) in 343 healthy, term newborns with a birth body weight of more than 2500 g. We then analyzed the association between body weight loss (BWL) and significant hyperbilirubinemia (total bilirubin more than 15 mg/dL) 72 hours after birth. Receiver operating characteristic curves were used to evaluate the appropriate cutoff BWL percentages on the first three days after birth for the prediction of neonatal hyperbilirubinemia 72 hours after birth.

**Results:**

A total of 115 (33.5%) neonates presented with significant hyperbilirubinemia 72 hours after birth, and the percentages of BWL on the first three days were all higher than those in the non-significant hyperbilirubinemia group (all *p* < 0.05). Breastfeeding was not statistically correlated with significant hyperbilirubinemia (*p=*0.86). To predict significant hyperbilirubinemia 72 hours after birth, receiver operating characteristic curve analysis showed that the optimum cutoff BWL percentages were 4.48% on the first day of life (sensitivity: 43%, specificity: 70%, positive likelihood ratio [LR^+^]: 1.43, and negative likelihood ratio [LR^-^]: 0.82), 7.60% on day 2 (sensitivity: 47%, specificity: 74%, LR^+^: 1.81, LR^-^: 0.72), and 8.15% on day 3 (sensitivity: 57%, specificity: 70%, LR^+^: 1.92, LR^-^: 0.61) (all *p* < 0.05).

**Conclusions:**

BWL on the first three days after birth may be a predisposing factor for neonatal hyperbilirubinemia, and may also serve as a helpful clinical factor to prevent significant hyperbilirubinemia 72 hours after birth. The optimal BWL cutoff percentages on the first three days after birth presented in this study may predict hyperbilirubinemia and indicate the need for supplementary feeding.

## Background

Sixty to 80% of healthy, full-term infants are expected to present with idiopathic neonatal jaundice in the initial postnatal period [1]. Idiopathic neonatal jaundice is attributed to an increased breakdown of heme, immature liver function, low amount of intestinal bacteria, increased enterohepatic circulation of bilirubin and inadequate intake [2]. Indicators of inadequate intake include 4 to 6 thoroughly wet diapers in 24 hours, and the passage of 3 to 4 stools per day by the fourth day. In addition, the stools of adequately breastfed infants should change from meconium to a mustard yellow, mushy stool by the third to fourth day. These assessments help to identify breastfed infants who are at risk of dehydration because of inadequate intake, however it is relatively subjective due to individual differences [3]. Compared to other methods, body weight loss (BWL) percentage is an objective and useful tool that may indicate when interventions such as supplemental feeding should be considered.

Previous studies have suggested that 7% to 10% BWL by day 3 in fully breastfed infants is abnormal neonatal BWL [4,5]. However, there are conflicting opinions about what constitutes normal neonatal BWL, and about when supplemental feeding should be considered to prevent significant hyperbilirubinemia. The aim of this study was to analyze the optimum cutoff values of BWL percentages in the first three days after birth to predict neonatal hyperbilirubinemia 72 hours after birth.

## Methods

### Patient population

We conducted a retrospective chart review of all neonates with a gestational age of more than 37 weeks and a birth body weight (BW) of more than 2500 g from January 2007 to December 2008 at a medical hospital. The exclusion criteria were a history of birth trauma, cephalohematoma, instrumental delivery, exposure to certain medications (mother or baby), history of gestational diabetes, early onset hyperbilirubinemia (less than 48 hours), pathological neonatal jaundice (including hemolysis, glucose-6-phosphate dehydrogenase deficiency, congenital infections, and congenital hypothyroidism), prenatal asphyxia, major organ anomalies, small for gestational age infants and infants who were exclusively formula fed.

In total, 166 neonates were excluded due to pathological neonatal jaundice (n = 42), early onset hyperbilirubinemia (n = 10), neonatal infections (n = 37), cephalohematoma (n = 7), prenatal asphyxia (n = 3), major organ anomalies (n = 2), and 65 who were discharged home within 2 days after birth. Three hundred and forty-three newborns were enrolled in further analysis. Gestational age was not analyzed, as the gestational age of all infants in the study was greater than 37 weeks, and most infants were around 39 to 40 weeks.

The study was approved by the institution’s Human Subjects Review Committee of Asia University in central Taiwan.

### Methods of analysis

The following variables were analyzed: gender, gestational age, birth BW, BW on the first day (24 hours) after birth (day 1), BW on the second day (48 hours) after birth (day 2), BW on the third day (72 hours) after birth (day 3), BWL/percentage on day 2 and day 3, feeding style (exclusively breast fed or mixed feeding), delivery method (normal spontaneous delivery or cesarean section), and total bilirubin level on day 3.

Exclusive breast feeding is the optimal initial feeding style for infants according to the WHO treatment guidelines. However, when there was insufficient breast milk formula was given, and the feeding style was classified as mixed feeding.

Transcutaneous total bilirubin was routinely checked in all infants using a transcutaneous device (BiliChek, Respironics, USA), and total serum bilirubin (TSB) was checked by heel stick for blood sampling (direct spectrophotometric method) when the infants presented with a transcutaneous bilirubin level of more than 15 mg/dL. The infants were divided into two groups: the significant hyperbilirubinemia group (total bilirubin level greater than 15 mg/dL 72 hours after birth) [6,7], and the non-significant hyperbilirubinemia group (total bilirubin level ≤ 15 mg/dL 72 hours after birth). The infants in the significant hyperbilirubinemia group were admitted for phototherapy. The correlations between BWL percentage within the first 3 days and the total bilirubin level 72 hours after birth were analyzed separately. We further analyzed the correlations between the TSB level and related clinical parameters.

### Statistical analysis

All statistical analyses were performed using Yate’s correction of contingency, *t*-test, Mann–Whitney U test, Spearman’s rank correlation, and receiver operating characteristic (ROC) curves. Because some data did not meet the normal assumption after a normal distribution test of the continuous variables, Spearman’s rank correlation was chosen. The results of the descriptive analyses of the independent variables were reported as percentages and mean ± standard deviation (SD). The differences between the groups were presented as 95% confidence intervals (CIs). Probability levels less than 0.05 were considered to be significant. Interobserver agreements in analyzing the scores of the two scoring systems were calculated by kappa statistics. We also examined the test parameters, including sensitivity, specificity, area under the ROC curve (AUC), positive likelihood ratio (LR^+^), and negative likelihood ratio (LR^–^) at the various cutoff values. The AUC, calculated using the trapezoidal rule, was considered as a global measure of the diagnostic value of that parameter. LR^+^ and LR^–^ were calculated for the best cutoff values. The criterion value indicated the value corresponding to the highest accuracy (minimal false-negative and false-positive results). Statistical analyses were performed using SPSS software (version 15.0; SPSS Inc., Chicago, IL, USA).

## Results

During the study period, 343 neonates with a gestational age of more than 37 weeks and a birth BW above 2500 g were analyzed. The mean birth BW was 3119 ± 352 g, and the mean BWL percentage on day 3 was 7.07 ± 2.82%. The mean TSB level 72 hours after birth was 13.39 ± 3.12 mg/dL. In addition, 115 neonates (33.5%) had levels of TSB higher than 15 mg/dL 72 hours after birth.

Table 1 lists the differences in variables between the significant and non-significant hyperbilirubinemia groups. There was no difference in birth BW between the two groups. The BWL percentages within the first 3 days after birth all showed a significant correlation with significant hyperbilirubinemia 72 hours after birth, and especially the BWL percentages on day 2 and day 3 (p < 0.001). The mean TSB level 72 hours after birth in the non-significant hyperbilirubinemia group was 11.67 ± 2.21 mg/dl, and 16.8 ± 1.36 mg/dl in the significant hyperbilirubinemia group. There were no significant correlations between different methods of feeding and delivery and significant hyperbilirubinemia 72 hours after birth.

**Table 1:** Differences between the two hyperbilirubinemia groups

	**Non-significant hyperbilirubinemia (n = 228)**	**Significant hyperbilirubinemia (n = 115)**	***P value***
Female	120(52.6%)	64(55.7%)	0.596^a^
Male	108(47.4%)	51(44.3)	
BW	3119.45 ± 356.43	3119.3 ± 344.46	0.935^c^
Day 1 BW (g)	3006.05 ± 344.10	2990.09 ± 335.45	0.746^c^
Day 2 BW (g)	2922.46 ± 335.86	2890.09 ± 329.5	0.423^c^
Day 3 BW (g)	2919.08 ± 339.02	2857.87 ± 331.55	0.132^c^
Day 1 BWL percentage	3.62 ± 1.95	4.14 ± 1.80	0.014^c^
Day 2 BWL percentage	6.29 ± 2.21	7.37 ± 1.82	< 0.001^c^
Day 3 BWL percentage	6.4 ± 2.73	8.4 ± 2.54	< 0.001^c^
72 hours bilirubin (mg/dL)	11.67 ± 2.21	16.8 ± 1.36	< 0.001^c^
Delivery methods			
NSD	136(59.60%)	75(65.20%)	0.377^b^
Cesarean section	92(40.40%)	40(34.80%)	
Feeding methods			
Exclusively breastfed	169(74.10%)	87(75.70%)	0.860^b^
Mixed feeding	59(25.90%)	28(24.30%)	

Data shown as mean ± SD or N(%).

^a^*P*-value by Chi-square test.

^b^*p*-value by Yate’s correction of contingency.

^c^*p*-value by Mann–Whitney U test.

BW: bodyweight; BWL: bodyweight loss; NSD: normal spontaneous delivery.

The strength of correlation among the BWL percentages within the first three days was identified by Spearman’s rank correlation coefficients (Table 2). The BWL percentages on day 1 and day 2 had a weak positive correlation with the TSB levels 72 hours after birth, while the BWL percentage on day 3 revealed a highly positive correlation (r = 0.407). In addition, the BWL percentage on day 3 was strongly correlated to the BWL percentage on day 2, while the BWL percentage on day 2 was only moderately correlated to the BWL percentage on day 1.

**Table 2:** Correlation between bilirubin level 72 hours after birth and bodyweight loss percentage within the first 3 days

		**72 hours Bil-T (mg/dl)**	**Day 1 BWL percentage**	**Day 2 BWL percentage**	**Day 3 BWL percentage**
**72 hours Bil-T (mg/dl)**	r	1			
	*p*-value				
	n	343			
**Day 1 BWL percentage**	r	0.149	1		
	*p*-value	0.006 **			
	n	343	343		
**Day 2 BWL percentage**	r	0.305	0.653^Ψ^	1	
	*p*-value	< 0.0001**	< 0.0001**	.	
	n	343	343	343	
**Day 3 BWL percentage**	r	0.407^Ψ^	0.355	0.751^ΨΨ^	1
	*p*-value	< 0.0001**	< 0.0001**	< 0.0001**	.
	n	343	343	343	343

Correlation analysis by Spearman’s rank correlation.

*:P < 0.05 **:P < 0.01.

^Ψ^0.4 < r < 0.7 ^ΨΨ^r > 0.7.

BW: bodyweight; BWL: bodyweight loss; Bil-T: total bilirubin level.

**Figure 1:**
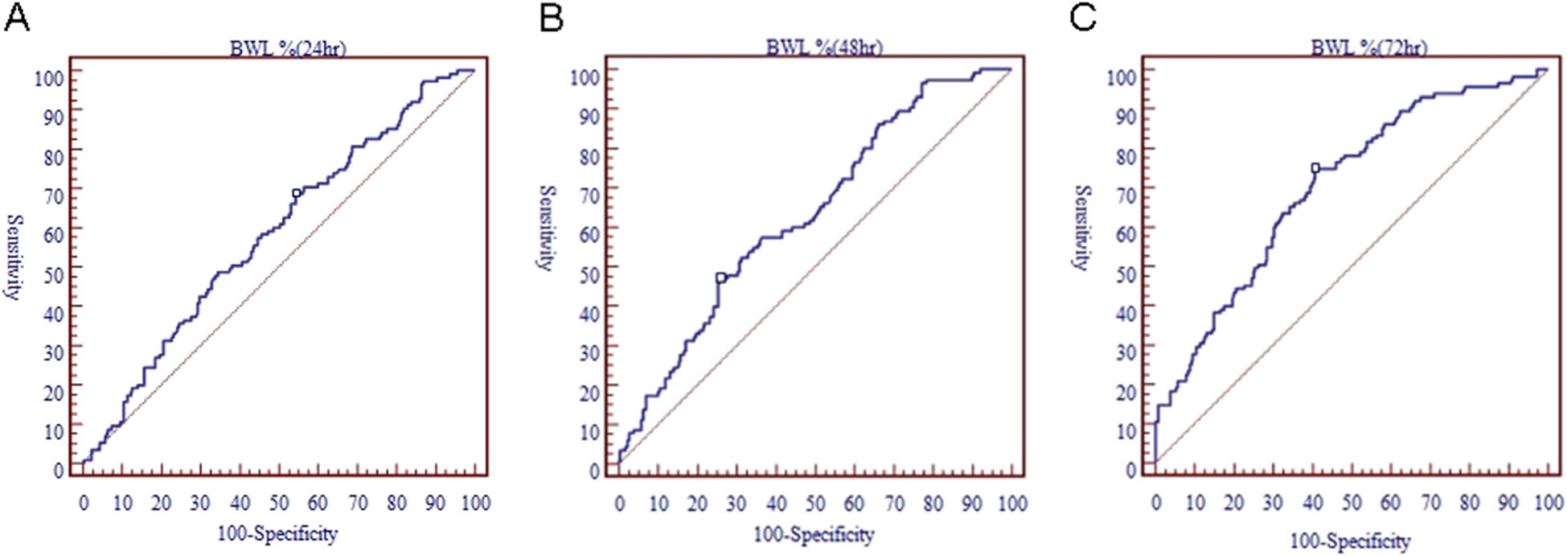
ROC curves of bodyweight loss percentage on day 1 (A), day 2 (B), and day 3 (C) to predict significant hyperbilirubinemia 72 hours after birth.

**Figure 2:**
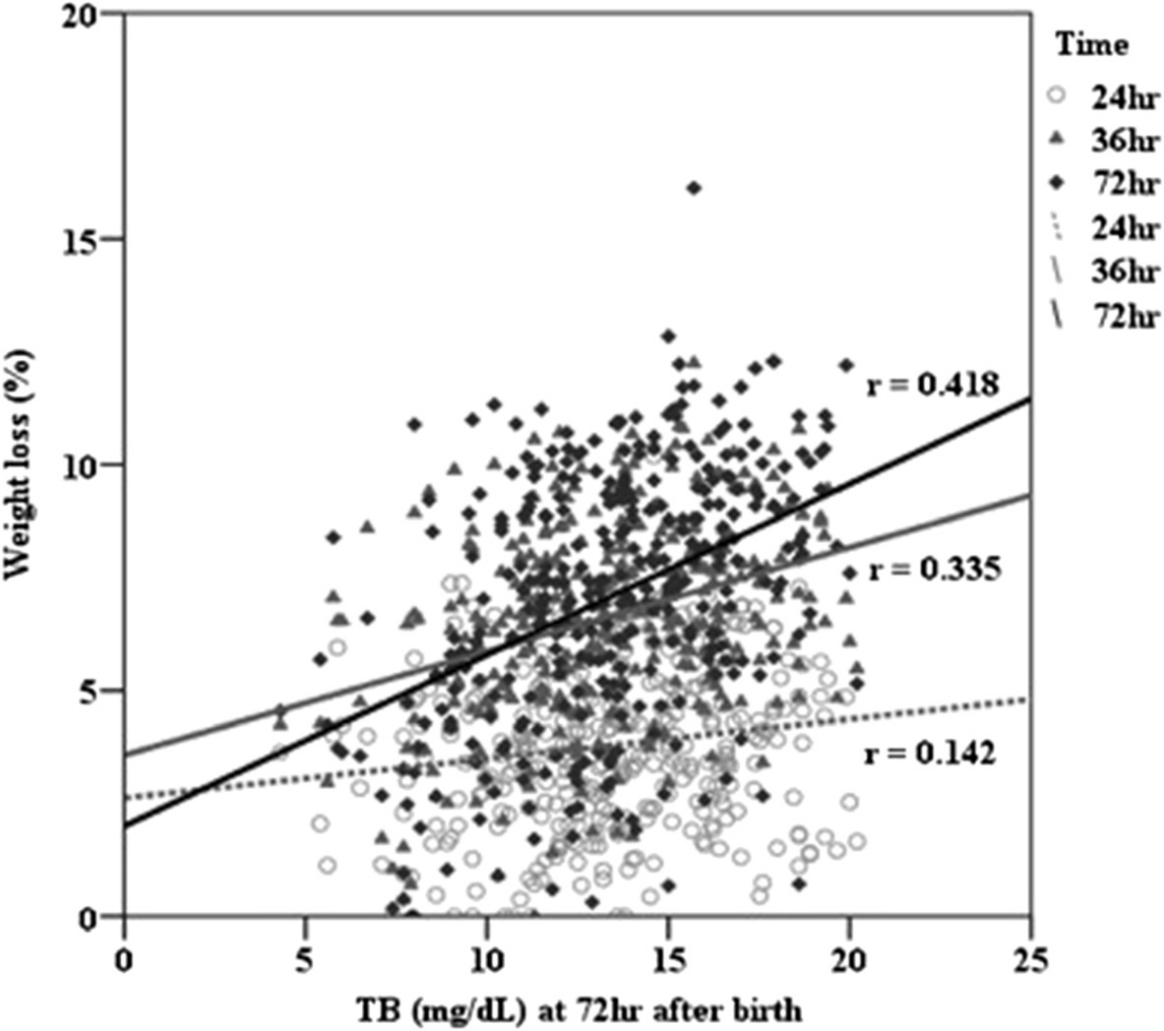
Analysis of correlation between body weight loss percentage (%) at 24, 48, and 72 hours after birth and total bilirubin levels (mg/dL) at 72 hours after birth.

Table 3 lists the upper and lower limits of the BWL percentage within the first 3 days in predicting significant hyperbilirubinemia 72 hours after birth by ROC analysis and the ROC curves are shown in Figure 1(A, B,C). The BWL percentage on day 3 had an AUC of more than 0.7. The neonates with BWL percentages less than 0.4% on day 1, less than 2.9% on day 2, and less than 0.6% on day 3 did not get significant hyperbilirubinemia 72 hours after birth. The infants with BWL percentages more than 10.2% on day 1, more than 10.9% by day 2, and more than 11.3% by day 3 had significant hyperbilirubinemia 72 hours after birth. In addition, a BWL percentage of 7.60% by day 2, and 8.15% by day 3 appeared to be the optimum cutoff points in predicting hyperbilirubinemia 72 hours after birth (Table 4). The correlation between body weight loss percentage within the first 3 days and total bilirubin levels at 72 hours after birth was performed by Pearson Correlation Coefficient and shown in Figure 2.

**Table 3:** The upper and lower limits of body weight loss percentage within the first three days to predict significant hyperbilirubinemia 72 hours after birth

**Day**	**BWL percentage**	**Sensitivity (%)**	**Specificity (%)**	**LR**^**+**^	**LR**^**-**^	**AUC**	**95% CI**	**P-value**
Day 1	>0.431%	100.00	4.39	1.05	0.00	0.581	0.527-0.634	0.014
	>10.19%	0.00	100.00	-	1.00			
Day 2	>2.97%	100.00	7.89	1.09	0.00	0.632	0.579-0.684	<0.001
	>10.93%	0.87	100.00	-	0.99			
Day 3	>0.60%	100.00	2.63	1.03	0.00	0.703	0.652-0.751	<0.001
	>11.33%	10.43	100.00	-	0.90			

BWL: bodyweight loss; LR: likelihood ratio; AUC: area under curve; CI: confidence interval.

**Table 4:** Optimal cutoff points of bodyweight loss percentage within the first three days to predict significant hyperbilirubinemia 72 hours after birth

**Day**	**BWL percentage**	**Sensitivity (%)**	**Specificity (%)**	**LR**^**+**^	**LR**^**-**^	**AUC**	**95% CI**	***P *value**
Day 1	>4.48%	42.61	70.18	1.43	0.82	0.581	0.527-0.634	0.014
Day 2	>7.60%	46.96	74.12	1.81	0.72	0.632	0.579-0.684	<0.001
Day 3	>8.15%	57.39	70.18	1.92	0.61	0.703	0.652-0.751	<0.001

BWL: bodyweight loss; LR: likelihood ratio; AUC: area under curve; CI: confidence interval.

## Discussion

Breastfeeding offers many benefits for neonates including host protection, better developmental outcomes, and decreased rates of infectious diseases, infant death syndrome, overweight and even asthma [8-12]. However, inadequate breastfeeding may cause excessive weight loss during the first postpartum days and may be associated with hyperbilirubinemia [13,14]. Breastfeeding has been thought to be a risk factor for significant hyperbilirubinemia since the 1960s [15-17], and breastfed infants have been shown to experience a maximum BWL by day 3 with a BWL of 6.6% compared to 3.5% for formula fed neonates [18]. This finding is believed to be attributable to the different components of mixed feeding, and may be because infants who receive mixed feeding have a poorer intake than those who are breastfed. In the current study, the healthy and term neonates who presented with significant hyperbilirubinemia 72 hours after birth had a BWL percentage of 8.4 ± 2.54% by day 3 compared to 6.4 ± 2.73% in the non-significant hyperbilirubinemia group. This finding is similar to previous studies in which 33% of 86 neonates with hyperbilirubinemia suffered from severe BWL of more than 10% [19], and 25% of 874 newborns with hyperbilirubinemia suffered a BWL of 8.96 ± 1.99% [20].

Even though many reports support the idea that breastfeeding induces severe hyperbilirubinemia, we found that breastfeeding was not statistically correlated with significant hyperbilirubinemia, thereby supporting the use of breast milk. A previous study reported that a BWL of more than 7% was an independent risk factor for early neonatal jaundice, and that infants with a BWL of more than 7% had a 1.4-fold increased risk of jaundice [21]. In the current study, the BWL percentages in the first three days after birth were all statistically significantly correlated with significant hyperbilirubinemia 72 hours after birth. In particular, the BWL percentage on day 3 was a better predictive factor for significant hyperbilirubinemia 72 hours after birth compared to day 1 and day 2. Moreover, regardless of being exclusively breastfed or mixed fed, the BWL within the first three days seemed to be influenced by the BWL on the previous day. This not only emphasizes the importance of BWL percentage on day 2 and day 3, but also suggests that the infants with a high BWL percentage on day 1 can still avoid further significant hyperbilirubinemia with optimal intervention. In this study, the order of birth (first born vs. second or third) was not analyzed since although the experience with breast feeding may influence the timing of giving extra formula milk and even the percentage of weight loss, the BWL was still the most objective factor regardless of how many babies the mother had.

We determined two cutoff points for each BWL percentage from day 1 to day 3 in order to be more easily applicable in a clinical setting, and to provide a point as to when significant hyperbilirubinemia 72 hours after birth can be ruled in or out. Based on the results of ROC analysis, the neonates with a BWL percentage on day 1 of less than 0.43% did not progress to significant hyperbilirubinemia 72 hours after birth, while a BWL percentage of more than 10.23% on day 1 led to significant hyperbilirubinemia 72 hours after birth. In addition, the neonates with BWL percentages less than 2.97% on day 2 and less than 0.60% on day 3 did not progress to significant hyperbilirubinemia 72 hours after birth, while those with BWL percentages of more than 10.93% on day 2 and 11.33% on day 3 did progress to significant hyperbilirubinemia 72 hours after birth. Primary care pediatricians should pay more attention to the patients in the indeterminate zone because it is not easy to predict significant hyperbilirubinemia 72 hours after birth in such cases. In addition, the physicians should attempt to persuade family members to increase the oral intake for these neonates to prevent further dehydration-induced significant hyperbilirubinemia 72 hours after birth. Based on our results, BWL percentage cutoff values of 4.48% on day 1, 7.60% by day 2, and 8.15% by day 3 were useful in predicting significant hyperbilirubinemia 72 hours after birth. We believe that this may be a helpful clinical tool when evaluating neonates with physical dehydration on the first three days after birth.

For non-pathological hyperbilirubinemia infants, dehydration is thought to be the major factor [1-5]. However, there seemed to be low accuracy when measured by the AUC curve with BWL at 24, 48, and 72 hours. In addition, the positive and negative likelihood ratios were very close to 1, suggesting that they had low practical significance. The cutoff values we propose are based on higher specificity and higher positive likelihood ratio, as the purpose was to prevent further significant hyperbilirubinemia. Accordingly, although BWL may not be an ideal predictor for significant hyperbilirubinemia, the results may be useful for physicians and parents as an indication of when extra feeding should be added.

There are some limitations to the present study. First, we used two different methods of measuring total bilirubin. Although the accuracy of a transcutaneous device has been proven, there may have been some deviation between the two methods. Second, maternal and neonatal risk factors for hyperbilirubinemia were not addressed (time of cord clamping, diabetic mothers, information on the order of birth in each group, previous siblings with significant jaundice and preterm infants). Third, the difficulty in assessing the adequacy of intake in breastfeeding and the variable timing of shifting to mixed feeding made the line between two feeding groups more blurred. Fourth, the major limitation may have been that we didn’t enroll more patients in the study. Finally, the study was performed in Taiwan and the cutoff values may not necessarily be applicable to other regions of the world.

## Conclusion

In conclusion, BWL on the first three days after birth may be a predisposing factor for neonatal hyperbilirubinemia, and may also serve as a helpful factor to prevent significant hyperbilirubinemia 72 hours after birth. The optimal BWL cutoff percentages on the first three days after birth presented in this study may predict hyperbilirubinemia and indicate the need for supplementary feeding.

## Abbreviations

BWL: Body weight loss; TSB: Total serum bilirubin; ROC: Receiver operating characteristic; BW: Bodyweight; AUC: Area under the ROC curve; LR+: Positive likelihood ratio; LR: Negative likelihood ratio.

## Competing interests

The authors declare that they have no competing interests.

## Authors’ contributions

WCY and LLZ reviewed the medical records, analyzed and interpreted the data, and drafted the manuscript; YCL and CHC interpreted the data and drafted the manuscript. YCF analyzed and interpreted the data. HPW designed and oversaw the study, interpreted the data, and revised the manuscript. All authors read and approved the final manuscript for publication.

## Pre-publication history

The pre-publication history for this paper can be accessed here:

http://www.biomedcentral.com/1471-2431/13/145/prepub
